# Bronchitis, and Kindred Diseases

**Published:** 1868-03

**Authors:** W. W. Hall

**Affiliations:** New York


### WIDLETON'S ADVERTISEMENT.

---

W. J. Widleton, No. 17 Mercer St., New York, has just published new Editions of "Health and Disease," of "Consumption," of "Sleep," (each $1.50—same by Mail.)

ALSO.

### BRONCHITIS, AND KINDRED DISEASES.

BY

W. W. HALL, A. M., M. D., NEW YORK.

There is no necessary reason why men should not generally live to the full age of three score years and ten, in health and comfort; that they do not do so, is because

They consume too much food, and too little pure air;

They take too much medicine, and too little exercise:

and when, by inattention to these things, they become diseased, they die chiefly, not because such disease is necessarily fatal, but because the symptoms which nature designs to admonish of its presence, are disregarded, until too late for remedy. And in no class of ailments are delays so uniformly attended with fatal results, as in affections of the Throat and Lungs. However terrible may have been the ravages of the Asiatic Cholera in this country, I know of no locality, where, in the course of a single year, it destroyed ten per cent. of the population. Yet, taking England and the United States together, twenty per cent. of the mortality is every year from diseases of the lungs alone; amid such a fearful fatality, no one dares say he shall certainly escape, while every one, without exception, will most assuredly suffer, either in his own person, or in that of some one near and dear to him, by this same universal scourge. No man, then, can take up these pages, who is not interested to the extent of life and death, in the important inquiry, *What can be done to mitigate this great evil?* It is not the object of this publication to answer that question; but to act it out; and the first great essential step thereto, is to impress upon the common mind, in language adapted to common readers, a proper understanding of the first symptoms of these ruthless diseases

Every reader of common intelligence and of the most ordinary observation, must know that countless numbers of people in every direction have been saved from certain death by having understood the premonitory symptoms of Cholera, and acting up to their knowledge. The physician does not live, who, in the course of ordinary practice, cannot point to a little army of the prematurely dead who have paid the forfeit of their lives by ignorance or neglect of the early symptoms of Consumptive disease. Perhaps the reader's own heart is this instant smitten at the sad recollection of similar cases in his own sphere of observation.

This book is not intended to recommend a medicinal preventive, or a patented cure for the diseases named on the title-page: it will afford no aid or comfort to those who hope, by its perusal, to save a doctor's fee, by a trifling tampering with their constitutions and their lives. Nor is it wished to make you believe, that if you come to me I will cure you. If you have symptoms of disease, I wish you to understand their nature first; and then to take advice from some regularly educated physician, who has done nothing to forfeit justly his honorable standing among his brethren, by the recommendation of secret medicines, patented contrivances or travelling lecturers for the cure of certain diseases. I may speak of persons in these pages, who had certain symptoms, and coming to me, were permanently cured. You may have similar symptoms, and yet I may be able to do you no good. I have sometimes failed to cure persons who had no symptoms at all. In other cases, where but a single symptom of disease existed, and it, apparently, a very trivial one, the malady has steadily progressed to a fatal termination, in spite of every effort to the contrary. The object of these statements is to have it understood, that I make no engagement to cure any thing or any body. The first great purpose is to enable you to understand properly any symptoms which you may have that point towards disease of the lungs; and when you have done so, to persuade you not to waste your time and money and health in blind efforts to remove them, by taking stuff, of which you know little, into a body of which you know less; but to go to a man of respectability and standing and experience—one in whom you have confidence, one who depends upon the practice of his profession for a living; describe your symptoms, according to your ability, place your health and life in his hands, and be assured that thus you and millions of others will stand the highest chance of attaining a prosperous, cheerful, and green old age. The rule should be universal, and among all classes, not only never to take an atom of medicine for anything, but not *to take anything as a* MEDICINE—not even a teaspoon of common syrup or French brandy, or a cup of red pepper tea, unless by the previous advice of a physician; because a spoonful of the purest, simplest syrup, taken several times a day, will eventually destroy the tone of the healthiest stomach: and yet any person almost would suppose that a little syrup "*could do no harm, if it did no good.*" A tablespoon of good brandy, now and then, is simple enough, and yet it has made a wreck and ruin of the health and happiness and hope of multitudes. If these *simple*, that is, *well-known things, in their purity*, are used to such results, it requires but little intelligence to understand that more speedy injuries must follow their daily employment, morning, noon, and night, when they are sold in the shape of "syrups," and "bitters," and "tonics," with other ingredients, however "*simple*" they, too, may be.

The common-sense reader will consider these sentiments reasonable and right, and think it a very laudable desire to diffuse information among the people as to the symptoms of dangerous, insidious, and wide-spreading diseases; but he will not be prepared for the information, that the publication of such a pamphlet as this will be considered "unprofessional" by some. But latitude must be allowed for difference of opinion; else, all progress is at an end. Whoever lends a helping hand to the diffusion of useful knowledge, is, in proportion, the benefactor of his kind. Whether it be useful for man to know the nature and first symptoms of a disease which is destined to destroy one out of every six in the country, is a question which each one must decide for himself. I believe that such an effort is useful, and hereby act accordingly. Experienced physicians constantly feel, in reference to persons who evidently have Consumption, that it is too late, because the application had been too long delayed. The great reason why so many delay, is because they "did not think it was anything more than a slight cold." In other words, they were entirely ignorant of the difference between the cough of a common cold and the cough of Consumption, and the general symptoms attendant on the two. It is not practicable for all to study medicine, nor is it to be expected that for every cough one has, he shall go to the expense of taking medical advice; it therefore seems to me the dictate of humanity to make the necessary information more accessible, and I know of no better way to accomplish this object than by the general distribution of a tract like this: and when I pretend to no new principle of cure, no specific, and no ability of success, beyond what an entire devotion to one disease may give any ordinary capacity, no further apology is necessary.

### THROAT-AIL, or Laryngitis, pronounced *Lare-in-*GEE-*tis*, is an affection of the top of the windpipe, where the voice-making organs are, answering to the parts familiarly called "Adam's Apple." When these organs are diseased, the voice is impaired, or "*there is something wrong about the swallow.*"

### BRONCHITIS, pronounced *Bron-*KEE-*tis*, is an affection of the *branches* of the windpipe, and in its first stages is called a common cold.

### CONSUMPTION

is an affection, not of the *top* or *root* of the windpipe, for that is *Throat-Ail;* not of the *body* of the windpipe, for that is *Croup;* not of the *branches* of the windpipe, for that is *Bronchitis;* but it is an affection of the lungs themselves, which are millions of little air cells or bladders, of various sizes, from that of a pea downwards, and are at the *extremities* of the branches of the windpipe, as the buds or leaves of a tree are at the extremity of its branches.

### WHAT ARE THE SYMPTOMS OF THROAT-AIL?

The most universal symptom is an impairment of the voice, which is more or less hoarse or weak. If there is no actual want of clearness of the sounds, there is an instinctive clearing of the throat, by swallowing, hawking, or hemming; or a summoning up of strength to enunciate words. When this is continued for some time, there is a sensation of tiredness about the throat, a dull heavy aching, or general feeling of discomfort or uneasiness, coming on in the afternoon or evening. In the early part of the day, there is nothing of the kind perceptible, as the voice-muscles have had time for rest and the recovery of their powers during the night. In the beginning of this disease, no inconvenience of this kind is felt, except some unusual effort has been made, such as speaking or singing in public; but as it progresses, these symptoms manifest themselves every evening; then earlier and earlier in the day, until the voice is clear only for a short time soon in the morning; next, there is a constant hoarseness or huskiness from week to month, when the case is most generally incurable, and the patient dies of the common symptoms of Consumptive disease.

In some cases, the patient expresses himself as having a sensation as if a piece of wool or blanket were in the throat, or an aching or sore feeling, running up the sides of the neck towards the ears. Some have a burning or raw sensation at the little hollow at the bottom of the neck; others, about Adam's Apple; while a third class speak of such a feeling or a pricking at a spot along the sides of the neck. Among others, the first symptoms are a dryness in the throat after speaking or singing, or while in a crowded room, or when waking up in the morning. Some feel as if there were some unusual thickness or a lumpy sensation in the throat, at the upper part, removed at once by swallowing it away; but soon it comes back again, giving precisely the feelings which some persons have after swallowing a pill.

Sometimes, this frequent swallowing is most troublesome after meals. Throat-Ail is not like many other diseases, often getting well of itself by being let alone. I do not believe that one case in ten ever does so, but on the contrary, gradually grows worse, until the voice is permanently husky or subdued; and soon the swallowing of solids or fluids becomes painful, food or drink returns through the nose, causing a feeling of strangulation or great pain. When Throat-Ail symptoms have been allowed to progress to this stage, death is almost inevitable in a very few weeks. Now and then a case may be saved, but restoration here is almost in the nature of a miracle.

### WHAT ARE THE SYMPTOMS OF BRONCHITIS?

Bronchitis is a bad cold, and the experience of every one teaches what its symptoms are. The medical name for a cold is *Acute Bronchitis;* called *acute*, because it comes on at once, and lasts but a short time—a week or two generally. The ailment that is commonly denominated *Bronchitis*, is what physicians term *Chronic Bronchitis;* called *chronic*, because it is a long time in coming on, and lasts for months and years instead of days and weeks. It is not like Throat-Ail, or Consumption, which have a great many symptoms, almost any one of which may be absent, and still the case be one of Throat-Ail, or Consumption; but Bronchitis has three symptoms, every one of which are present every day, and together, and all the time, in all ages, sexes, constitutions, and temperaments. These three universal and essential symptoms are—

1st. A feeling of fullness, or binding, or cord-like sensation about the breast.

2d. A most harassing cough, liable to come on at any hour of the day or night.

3d. A large expectoration of a tough, stringy, tenacious, sticky, pearly or greyish-like substance, from a tablespoon to a pint or more a day. As the disease progresses, this becomes darkish, greenish, or yellowish in appearance; sometimes all three colors may be seen together, until at last it is uniformly yellow, and comes up without much effort, in mouthfuls, that fall heavily, without saliva or mucus. When this is the case, death comes in a very few weeks or—days.

### WHAT ARE THE SYMPTOMS OF CONSUMPTION?

A gradual wasting of breath, flesh, and strength are the three symptoms, progressing steadily through days and weeks and months, which are never absent in any case of true, active, confirmed Consumptive disease that I have ever seen. A man may have a daily cough for fifty years, and not have Consumption. A woman may spit blood for a quarter of a century, and not have Consumption. A young lady may breathe forty times a minute, and have a pulse of a hundred and forty beats a minute, day after day, for weeks and months together, and not have Consumption; and men and women and young ladies may have pains in the breast, and sides, and shoulders, and flushes in the cheeks, and night sweats, and swollen ankles, and yet have not an atom of Consumptive decay in the lungs. But where there is a slow, steady, painless decline of flesh and strength and breath, extending through weeks and months of time, Consumption exists in all persons, ages, and climes, although at the same time sleep, bowels, appetite, spirits, may be represented as good. Such, at least, are the results of my own observation.

The great, general, common symptoms of Consumption of the Lungs are night and morning cough, pains about the breast, easily tired in walking, except on level ground, shortness of breath on slight exercise, and general weakness. These are the symptoms of which Consumptive persons complain, and as they approach the grave, these symptoms gradually increase.

### HOW DOES A PERSON GET THROAT-AIL?

A woman walked in the Park, in early spring, until a little heated and tired; then sat down on a cold stone. Next day, she had hoarseness and a raw burning feeling in the throat, and died within the year.

A man had suffered a great deal from sick headache; he was advised to have cold water poured on the top of his head: he did so; he had headache no more. The throat became affected; had frequent swallowing, clearing of throat, falling of palate, voice soon failed in singing, large red splotches on the back part of the throat, and white lumps at either side; but the falling of the palate and interminable swallowing were the great symptoms, making and keeping him nervous, irritable, debilitated, and wretched. He was advised to take off the uvula, but would not do it. Had the nitrate of silver applied constantly for three months. Tried homœopathy. After suffering thus two years, he came to me, and on a subsequent visit, said. "It is wonderful, that for two years I have been troubled with this throat, and nothing would relieve it, and now it is removed in two days." That was four months ago. I saw him in the street yesterday. He said his throat gave him no more trouble; that he had no more chilliness, and had never taken a cold since he came under my care, although formerly "it was the easiest thing in the world to take cold."

A merchant (1002) slept in a steamboat state-room in December, with a glass broken out; woke up next morning with a hoarseness and sore throat; for several months did nothing, then applied to a physician. Counter-irritants were employed without any permanent effect. At the end of four years he came to me with "a sort of uneasy feeling about the throat, more at times than others; not painful; sometimes a little hoarseness, with frequent inclination to swallow, or clear the throat. At the little hollow at the bottom of the neck, just above the top of the breast-bone, there was a feeling of pressure, stricture, or enlargement—no pain, but an unpleasant sensation, sometimes worse than at others. It is absent for days at a time, and then lasts for several hours a day." This case is under treatment.

A Clergyman (1012) has a hoarse, cracked, weak voice, easily tired in speaking; a raw sensation in the throat; and in swallowing has "*a fish-bony feeling.*" He had become over-heated in a public address, and immediately after its close started to ride across a prairie in a damp, cold wind in February. Had to abandon preaching altogether, and become a school teacher." This gentleman wrote to me for advice, and having followed it closely for eighteen days, reported himself as almost entirely well.

I greatly desire it to be remembered here, that in this, as in other cases of Throat-Ail, however perfectly a person may be cured, the disease will return as often as exposure to the causes of it in the first place is permitted to occur. No cure, however perfect, will allow a man to commit with impunity such a thoughtless and inexcusable act as above named, that of riding across a prairie in February, in a damp, cold wind, within a few minutes after having delivered an excited address in a warm room. None of us are made out of India rubber or iron, but of flesh and blood and a reasonable soul, subject to wise and benevolent conditions and restrictions; and it is not to the discredit of physic or physicians, that being once cured, the disease should return as often as the indiscretion that originated it in the first instance is re-committed.

Three weeks ago, one of our merchants came to me with a troublesome tickling in the throat. At first it was only a tickling; but for some weeks the tickling compels a frequent clearing of the throat; and without a cough, each clearing or hemming brings up half a teaspoon-ful of yellow matter, with some saliva. On looking into his throat, the whole back part of it was red, with still redder splotches here and there—epiglottis almost scarlet. On inquiry, I found he had for years been a chewer of tobacco; then began to smoke; would day after day smoke after each meal, but especially after tea would consume half a dozen cigars. In time, the other naturally consequent steps would have been taken—Consumption and the grave. Among other things, I advised him to abandon tobacco absolutely and at once. In two weeks he came again. Throat decidedly better; in every respect better, except that he, in his own opinion, "had taken a little cold," and had a constant slight cough—not by any means a trifling symptom. Let the reader learn a valuable lesson from this case. This gentleman had the causes of cough before; he found that smoking modified the tickling, and taking this as an indication of cure, he smoked more vigorously, and thus suppressed the cough, while the cause of it was still burrowing in the system and widening its ravages. It will require months of steady effort to arrest the progress of the disease, and he may consider himself fortunate—more so than in any mercantile speculation he ever made—if he gets well at all. If he does get well, and returns to the use of tobacco, the disease will as certainly return as that the same cause originated it, for the following reason, as was stated in the *First Part:*—Throat-Ail is inflammation; that is, too much heat in the parts. Tobacco smoke being warm, or even hot, is drawn directly back against the parts already too much heated, and very naturally increasing the heat, aggravates the disease. Again, any kind of smoke—that of common wood—is irritating, much more that of such a powerful poison as tobacco.

—soothing, indeed, in its first transient effects, like many other poisons, but leaving behind it consequences more remote, but more destructive and enduring.

A gentleman, just married, with a salary for his services as secretary to a Southern house, applied to me to be cured of a sore throat. He was permanently hoarse; swallowing food was often unendurably painful, besides causing violent paroxysms of cough. He said he knew no cause for his complaint, except that he had smoked very freely. On inquiry, I found that for the last two years he had used, on an average, about "a dozen cigars every day; perhaps more." He died in six weeks.

In several instances, persons have applied to me who had been advised to take brandy freely for a throat affection. Such advice is warranted by no one principle in medicine, reason, or common sense. Were I to give it, I should feel myself justly liable to the charge of being an ignorant man or a drunkard. The throat is inflamed; inflammation is excitement; brandy and tobacco both excite, inflame the whole body; that is why they are used at all. The throat partakes of its portion of the excitement, when the throat, body, and the man, all the more speedily go to ruin together. I have in my mind, while writing these lines, the melancholy history of two young men—one from Kentucky, the other from Missouri—who were advised "to drink brandy freely, three times a day, for throat complaint." One of these became a drunkard, and lost his property, and within another year he will leave an interesting family in penury, disgrace, and want. The other was one of the most high-minded, honorable young men I have lately known. He was the only son of a widow, and she was rich. He came to see me three or four times, and then stated that he had concluded to try the effects of a little brandy at each meal. A few weeks afterwards he informed me, that as he was constantly improving, he thought that the brandy would certainly effect a cure. Within seven months after his application to me, he had become a regular toper; that is, he had increased the original quantity allowed, of a tablespoon at each meal, to such an amount, that he was all the time under the influence of liquor. His business declined; he spent all his money; and secretly left for California, many thousand dollars in debt, and soon after died. The person who advised him is also now a confirmed drunkard; but in his wreck and ruin, still a great man.

A gentleman from a distant State wrote to me some months ago for advice as to a throat affection. He is a lawyer of note already, and of still higher promise, not yet having reached the prime of life. By earnest efforts as a temperance advocate, in addition to being a popular pleader at the bar, his voice became impaired with cough, spitting of blood, matter expectoration, diarrhœa, debility, and general wasting. He was induced to drink brandy with iron, but soon left off the iron and took the brandy pure. The habit grew upon him; he *sometimes* stimulated to excess, according to his own acknowledgment; his friends thought there was no interval, and gave him up as a lost man to themselves, his family, and his country; but in time the virulence of the disease rose above the stimulus of the brandy, and in occasional desperation he resorted to opium. He subsequently visited the water cure, gained in flesh and strength, and was hopeful of a speedy restoration; but he took "an occasional cigar" —the dryness in the throat, hoarseness, pain or pressure, and soreness still remained! He left the water cure, and in a few months wrote to me, having, in addition to the above throat symptoms, a recent hæmorrhage, constipation, pains in the breast, nervousness, debility, variable appetite, and daily cough. Within two months, he has become an almost entirely new man, requiring no further advice.

Further illustrations of the manner in which persons get Throat-Ail, may be more conveniently given in the letters of some who have applied to me, with the additional advantage of having the symptoms described in language not professional, consequently more generally understood.

### A PRESBYTERIAN CLERGYMAN.

(1059) "I have had for three years past a troublesome affection of the thorax, which manifests itself by frequent and prolonged hemming or clearing the throat, and swelling: both more frequent in damp weather, or after slight cold. General health very feeble, sleeplessness, waste of flesh, low spirits. Visited a water cure, remained two months, but my hemming and swallowing were not a whit improved. Touching with the nitrate of silver slightly makes the larynx sore. I have been always able to preach. It has never affected my voice until very recently. Two weeks ago I preached two long sermons, in a loud and excited voice, in one day. During the last discourse my voice became hoarse, and my hemming has become very bad; and there has been a slight break in my voice ever since. Hem, hem, hem, is the order of the day; clearing the throat is incessant, swallowing often, and a slight soreness of the larynx, particularly after a slight cold, or after several days' use of nitrate of silver, with a scarce perceptible break in the voice. These are my principal symptoms."

This case is under treatment.

### A LAWYER,

(1016) "aged thirty-seven. Have been liable, for several years past, in the fall, winter, and spring, to severe attacks of fever, accompanied with great debility, loss of flesh, appearing to myself and friends to be in the last stages of Consumption; in fact, the dread of it has been an incubus on me, paralyzing my energies and weighing down my spirits. In the summers, too, I have been subject to attacks of bilious fever and bilious colic. A year ago, I attended court soon after one of these attacks, and exerted myself a great deal. My throat became very sore, and I had hemorrhage—two teaspoons of blood and matter. My health continued feeble. I went last summer to a water cure, and regained my flesh and strength, but the weakness in my throat and occasional hoarseness continued all the time. Afterwards, by cold and exposure, I became worse, continued to have chills and fever and night sweats, accompanied by violent cough and soreness of the throat. I got worse; was reduced to a perfect skeleton, and had another hæmorrhage. Mucus would collect in the top of the throat, and was expectorated freely. I am still liable to colds. The seat of the disease seems to be at the little hollow in front at the bottom of the neck, just above the top of the breast-bone. At my last bleeding, the pain seemed to be in the region of Adam's-apple. The principal present symptoms are soreness in throat, dryness, pain on pressing it, and hoarseness; pulse from eighty to ninety in a minute; irregular appetite. These symptoms, together with my fear of Consumption, serve to keep me unhappy. I find myself constantly liable to attacks of cold, sneezing, running at the nose even in the summer time. My mother and sister have died of Consumption, as also two of my mother's sisters. Feet always cold; daily cough."

### OPINION OF THE CASE.

There is no Consumptive disease: it is impossible. No personal examination is needed to tell that. The foundation of all your ailments is a torpid liver and a weak stomach. If you are not cured, it will be your own fault.

The treatment of this case was conducted by correspondence, as he lived six hundred miles away, and therefore I had not the opportunity of a personal examination. Within a month he writes:—"I am gradually improving; feet warm; all pain has disappeared from the breast; appetite strong, regular, and good: pulse seventy-two; breathing eighteen; all cough has disappeared." At the end of two and a half months, no further advice was needed, as he wrote—"I have not written to you for a month, being absent on the circuit. I have not enjoyed better health for years than I have for the month. Weight increasing; no uneasiness or pain about my breast; pulse seventy-five; less in the morning. The only trouble I have is costiveness, from being so confined in court, and being away from home deprived of my regular diet. We were two weeks holding court, last of November, in a miserable room, the court-house having been recently burned; kept over-heated all the time. I made four or five speeches, and suffered no inconvenience whatever. I have no cough."

### A CLERGYMAN

(1024) called over two months ago, having had at first an ailment at the top of the throat, apparently above or near the palate. It soon descended to the region of Adam's-apple, and within a month it seemed to have located itself lower down the neck, giving a feeling as if there were an ulcer there, with a sense of fullness about the throat, hoarse after public speaking, lasting a day or two, with attacks every few weeks of distressing sick headache. As the disease seemed to be rapidly descending towards the lungs, a rigid, energetic treatment was proposed, and at the end of ten weeks he writes—"I take pleasure in introducing my friend, ——, to you. He has suffered many things, from many advisers, with small benefit. I have desired him to consult with you, hoping that he may have the same occasion to be grateful for the providence which leads him to you, which I feel that I myself have for that which guided me to your counsels. I suffer but little, very little from my throat, and confidently anticipate entire relief at no distant day, for all which I feel myself under great obligation both to your skill and to your kindness," &c.

### SICK HEADACHE

is a distressing malady, as those who are subject to it know full well, by sad experience. In this case, this troublesome affection had to be permanently removed before the throat ailment could be properly treated; when that was done, the throat itself was comparatively of easy management.

### A MERCHANT

(947) wrote to me from the South, complaining chiefly of

Bad cough, sometimes giving a croupy sound;
Throat has a raw, choking, dry, rasping feeling;
Soon as he goes to sleep, there is a noise or motion, as if he were going to cough;
Startled in sleep, by mouth filling with phlegm;
Expectoration tough, white, and sticky; darkish particles sometimes;
Flashes or flushes pass over him sometimes;
Sick stomach sometimes, acid often, wind on stomach oppresses him greatly;
A lumpy feeling in the throat;
On entering his house, sometimes falls asleep in his chair, almost instantly;
In walking home, at sundown, half a mile from his store, is completely exhausted;
Slightest thing brings on a cough; never eats without coughing;
If he swallows honey, it stings the throat;
Got a cold a month ago, which left the palate and throat very much inflamed;
Throat and tongue both sore;
A hooping, suffocative cough; can hear the phlegm rattle just before the cough begins;
A dry, rough feeling from the little hollow at the bottom of the neck up to the top of the throat.
One night after going to bed, began to cough, choke, suffocate; could not get breath, jumped out of bed, ran accross the room, struggled, and at length got breath, but was perfectly exhausted; could not speak for half an hour, without great difficulty.

In addition to his own description of the case, his wife writes—"Ten o'clock at Night.—I am no physician, nor physician's wife, but am his wife and nurse, and an anxious observer of his symptoms, and can see his throat inflamed behind the uvula. He says there is a lump somewhere, but he cannot tell where. Sometimes he thinks it is in the little hollow at the bottom of the neck, sometimes ju t above, and sometimes in or about the swallow. A recent cold has aggravated his symptoms. His cough to-day has been very frequent and loose. He has emaciated rapidly within a month, and is now a good deal despondent. As for myself, I feel as one who sees some fair prospect suddenly fading away. I had fondly hoped—oh! how ardently!—that he might be restored. If a knowledge of the fact would give any additional interest to the case, I will only say, he is one of the loveliest characters on earth. None in this community has a larger share of the respect and confidence of their acquaintance."

The opinion sent, for I have not seen this case, was as follows:—"The whole breathing apparatus, from the top of the windpipe to the extremity of its branches, is diseased; the lungs themselves are not at all affected by decay. Your whole constitution is diseased; and yet there is good ground for hope of life and reasonable health."

In three months this patient writes—"I am glad to inform you that I think I am still improving in health and strength. My bowels are sometimes disordered by eating melons and fruits; but I felt so much better that I thought I might indulge. Pulse sixty-five to seventy; an almost ravenous appetite." A month later he writes—"My health and strength are still improving; cough not very troublesome; increasing flesh," &c. I believe this gentleman now enjoys good health.

### A LADY.

(948) teacher of vocal music, writes—"There is a peculiar sensation in my throat for the last two months. Whenever I attempt to swallow, it feels as if something were in the way; a swelling under the jaws, a soreness on the sides of the throat, extending to the ears, and occasioning throbbing painfully. I have a dull aching at the top of my collar-bone, and an unpleasant sensation of weakness and heaviness in my chest; a bad taste in my mouth frequently. Have been regular, but have been afflicted for a few years past with sickness at the stomach and vomiting, attended occasionally with great pain for a few hours. During these attacks, the complexion changes to a livid hue. I have been very much troubled with dyspepsia. On recovering from the attacks above mentioned, I have experienced a feeling of weakness almost insupportable. Am very costive; and my spirits are greatly depressed. Within a day or two I have taken a violent cold, which has affected me with sneezing, running from the eyes and nose, together with a slight hoarseness. I was advised to apply caustic to the throat, and Croton oil to my neck, chest, and throat. I have since discontinued these, not having received any permanent benefit from them. On two occasions, from over-exertion at concerts and examinations, I was unable to speak a loud word, from hoarseness, for several days. I am extremely anxious to learn your opinion. In about two months my public concerts take place, and it is absolutely necessary that something should be done for me."

### OPINION.

Yours is general constitutional disease. There is no special cause of alarm. A weakened stomach, a torpid liver, a want of sufficient air and exercise, are the foundations of all your ailments, and by the proper regulation of these, you may expect to have good health and a stronger voice. You must have energy and patient perseverance in carrying out the prescriptions sent to you.

In one month this lady writes, and the letter is given to encourage others who may come under my care, to engage with determination and energy in carrying out the directions which may be given them. The reader may also see what great good a little medicine may do when combined with the judicious employment of rational means, which do not involve the taking of medicine or the use of painful and scarifying agencies and patent contrivances:—

"I began your prescriptions at once. Having followed them for some time, I was obliged to intermit them for a few days, in consequence of having to conduct a concert, besides having to travel by stage and railroad seventy or eighty miles. During this time, I was up every night until twelve o'clock, and was much exposed to the night air. On returning home, I re-commenced your directions, have made it a point to attend to them strictly, and have very seldom failed of doing so. In consequence of two omissions in diet, I suffered from headache, which disappeared when I observed your directions. My appetite is good; my food agrees with me. I sometimes feel dull and sleepy after dinner. I drop to sleep immediately. Seldom wake in the night. Sleep about seven hours, and generally feel bright and strong in the morning, when I take a brisk walk of two miles and a half; the same after six, P.M. My walks at first fatigued me considerably; generally, however, I have felt better and better from their commencement to their end, and have perspired very freely. The exercise I take seems rather to increase than diminish my strength. I have not been prevented from taking exercise from any dampness in the atmosphere. I have sometimes been exposed to the night air in going to church and other places, but without any perceptible injury. The means you advised produce a general glow, and invariably remove headache, which I sometimes have to a slight degree after dinner. I think my throat is better. There is no unpleasant feeling about it at present, except the difficulty in swallowing, and even that is better. Pulse sixty-seven."

I had for some time ceased to regard this energetic young lady as a patient, when she announces a new ailment, a difficulty at periodic times:—"I walked two miles every day, and every thing was going on well, until one evening after walking very fast, I sat awhile with a friend, in a room without fire, in November. The weather was chilly and damp; was unwell, suppressed; had a chill and incessant cough for several hours, ending in something like inflammation of the lungs."

These things were remedied, and she is now engaged in the active discharge of her duties. This last incident is introduced here to warn every reader, especially women, against all such exposures at all times, most especially during particular seasons. Such exposures, as sitting in rooms without fire, in the fall and spring, after active walking, have thrown stout strong men into a fatal consumption; and it is not at all to be wondered at that delicate women should lay the foundation of incurable disease in the same manner. I will feel well repaid for writing these lines, if but here and there a reader may be found to guard against such exposures. Our parlors and drawing-rooms are kept closed to the air and light for a great portion of the twenty-four hours, and unless the weather is quite cool there is no fire in them. Thus they necessarily acquire a cold, clammy dampness, very perceptible on first entering. A fire is not thought necessary, as visitors usually remain but a few minutes; but when the blood is warmed by walking in the pure air and the clear sunshine, it is chilled in a very short space of time, if the person is at rest, in the cold and gloom of a modern parlor, especially as a contemplated call of a minute is often unconsciously extended to half an hour, under the excitement of friendly greetings and neighborly gossip. There can be no doubt that thousands every year *catch their death of cold*, to use a homely but expressive phrase, in the manner above named. Young women, especially, cannot act thus with impunity. Men perish by multitudes every year by exposures of a similar character; walking or working until they become warm, then sitting in a hall or entry or a cold counting-room; or standing still at the wharf or at a street corner; or running to reach a ferry-boat until they begin to perspire, and then sitting still in the wind while the boat is crossing. It is by inattention to what may be considered *such trifling little things* that thousands of valuable lives are sacrificed every year.

### A YOUNG GENTLEMAN,

(950) from Washington City, complained of

Uneasiness at throat, caused by repeated colds; late hours, hot rooms;
Cough most of mornings—dry, tickling, hollow;
Expectoration a little yellow;
Bloody, streaked expectoration, six months ago;
Breathing oppressed, if sit or stoop long;
Take cold easy, in every way;
Throat has various feelings, tickling, heavy aching, raw, dry, from palate to depression;
Swallowing a little difficult at times;
Voice not much affected;
Headache, costive bowels, piles occasionally;
Pain about shoulder-blades and at their points;
Soreness under both ribs sometimes;
Pains in the breast—more of a soreness from the top of the breast-bone to the pit of the stomach;
Have been ailing fifteen months;
Father, mother, sister, uncle, aunt died of Consumption.

### OPINION.

You cannot have Consumption now: you are decidedly threatened with it. With proper attention, persevering and prompt, you may ward it off effectually, and live to the ordinary term of human life to those of your occupation. It is my opinion, that without this care, you will fall into settled disease within a year.

In two months, this gentleman called to see me for the first time. His lungs were working freely and fully, over the natural standard; pulse seventy-two; appetite good; bowels regular. I did not think he required any particular medical advice; and it is my present belief, that with proper attention to diet, exercise, and regular habits of life, his health will become permanently good.

952.

Took a severe cold last winter, which left a severe cough. Every morning the breast feels sore, until stirs about some. Pain in the left side, running through to the left shoulder blade, and between the shoulders; pain in the breast-bone, and in the centre of the left breast. Chief complaint is pain in the chest, left side, and a constant raising of frothy, thick, tough, and yellow matter, with frequent hawking, hemming, and clearing of the throat. Age 22.

### OPINION.

Your ailments are all removeable by diligent attention to the directions I may give you. I very much hope you will spare no pains in carrying them out most thoroughly. You certainly have not Consumptive disease.

He called upon me some months afterwards, when I saw him for the first time. He had nothing to complain of; pulse sixty; his lungs working freely and fully, being considerably above the natural standard; and as far as I know, he continues well to this day.

973.

"Am officer in a bank. Was at a fire during Christmas, seven months ago. Used my voice a great deal; began to be hoarse; very much so by morning. This lasted a week, and went off; but in three weeks there appeared to be something about the palate which wanted to come away. Throat seemed inflamed, and ever since then have had a clogging feeling in the throat, that does not affect my voice, unless I read aloud, when I soon become hoarse. Two days ago, spit up a spoonful of dark blood; never before or since. I have a binding sensation across the top of the breast, and three months since had a pain up and down the breast-bone. Have used iodide of potash; have had the throat pencilled, and then sponged with nitrate of silver, without benefit—pulse, one hundred and ten."

### OPINION.

Yours is a throat ailment, at the entrance of the windpipe—not as low down as the voice organs. There is very considerable active inflammation there. Your lungs are a little weakened, nothing more; the pains in the breast are not serious at all, and I see no obstacle to your entire recovery.

I received letter after letter from this young gentleman, stating that no perceptible benefit seemed to follow what I advised. He was encouraged to persevere, and finally his symptoms began to change, and then disappeared; and in two months from his first consultation he wrote me to say that he had steadily improved; pulse, permanently at sixty-five; expressing his obligations, &c. This case shows strikingly the advantage of perseverance.

### A CLERGYMAN

(844) wrote to me for advice in reference to a throat complaint. I prescribed, and had entirely forgotten the circumstance, when the following letter was received:—

"I began to follow your directions on the 4th day of May, not quite three months ago, and have adhered to them strictly ever since. I am evidently a great deal better. I have lost no flesh; although it is summer, my weight has not varied three pounds since I wrote to you; it is now one hundred and forty-nine pounds. My tonsils are diminished, and give me no uneasiness, except in damp weather. From my throat, which is now generally perfectly comfortable, I am continually bringing up a pearly substance. Sometimes it is perfectly clear, and like the pure white of an egg. But this is a mighty change. At first, I could not talk five minutes in the family circle. My throat was constantly tickling and burning; so that a mustard plaster, which took all the skin off my neck in front, was a comfort; but now I can talk as much as I wish, read a page or so aloud, and am almost tempted to sing a little."

### HOW DO PERSONS GET BRONCHITIS?

In the same manner as a common cold, for Bronchitis is a common cold protracted, settling not on the lungs, but on the branches of the windpipe, clogging them up with a secretion thicker than is natural; this adheres to the inside of the tube-like branches, and to a certain extent closes them: hence, but a small portion of air gets into the lungs. Nature soon begins to feel the deficiency, and instinctively makes extra effortsto obtain the necessary quantity, in causing the patient to draw in air forcibly instead of doing it naturally and without an effort. This forcible inspiration of external air drives before it the accumulating phlegm, and wedges it more compactly in a *constantly-diminishing tube*, until the passage is entirely plugged up. The patient makes greater efforts to draw in the air, but these plugs of mucus arrest it, and there is a feeling as if the air did not get down to its proper place, or as if it were stopped short, causing a painful stricture, or cord-like sensation, or as some express it, *a stoppage of breath*. If relief is not given in such cases, either by medicine judiciously administered, or by a convulsive nature of effort at a cough, which is a sudden and forcible expulsion of such air as happened to be *on the other side* of the plug, the patient would die; and they often do feel as if they could not possibly live an hour. This is more particularly a description of an attack of Acute Bronchitis. Chronic Bronchitis is but a milder form of the same thing, very closely allied in the sensations produced, if not indeed in the very nature of the thing, to what may be considered a kind of

### PERPETUAL ASTHMA, which may in most cases be removed and warded off for an indefinite time by the use of very little medicine, if the patient could be induced to have a reasonable degree of self-denial and careful perseverance.

### HOW DO PERSONS GET CONSUMPTION?

As they do most other diseases, by inattention, neglect, imposition on nature. Many persons have this disease hereditarily, but the same means which permanently arrest the progress of accidental Consumption will as often and as uniformly ward off, indefinitely, the effects and symptoms of the hereditary form, the essential nature of accidental and hereditary Consumption being the same. The treatment is also the same, except that in the accidental form it must be more prompt, more energetic; in the hereditary form it must be more mild, more persevering. I consider the latter, the less speedily and critically dangerous of the two.

### MISCELLANEOUS ITEMS.

A number of pages will be devoted to the illustration of a variety of topics connected with the general subject; all, however, will be of a practical character—at least, such is the intention.

CONSUMPTION IS THE OXIDATION OF THE EXUDATION CORPUSCLE. This corpuscle—*this little body*, this tubercle, this seed of Consumption—is an albuminous exudation, as minutely described on page 5, First Part, and being deficient in fatty matter, its elementary molecules cannot constitute nuclei, capable of cell development; therefore, these nuclei remain abortive, are foreign bodies in the lungs, and like all other foreign bodies there, cause irritation, tickling. This tickling is a cause of cough, as itching is a cause of scratching, both being instinctive efforts of nature to remove the cause of the difficulty. The oxidation—that is, the burning, the softening of this corpuscle or tubercle—gives yellow matter as a product, just as the burning—that is, the oxidation of wood—gives ashes as a product. Thus the yellow matter expectorated in Consumption is a sign infallible, that a destructive, consuming process is going on in the lungs, just as the sight of ashes is an infallible sign that wood or some other solid substance has been burned—that is, destroyed.

But why is it that this albuminous exudation, this tubercle, this exudation corpuscle, should lack this fatty matter, this oil, this carbon, which, did it have, would make it a healthy product, instead of being a foreign body and a seed of death?

Consumption is an error of nutrition. The patient has soliloquized a thousand times, "I sleep pretty well, bowels regular, and I relish my food, but somehow or other it does not seem to do me the good it used to. I do not get strong." The reason of this is, that the food is imperfectly digested, and when that is the case, acidity is the result, which is the distinguishing feature of Consumptive disease. This **excess** of acid in the alimentary canal dissolves the albumen of the food, and carries it off into the blood in its dissolved state, making the whole mass of blood in perfect impure, thick, sluggish, damming up in the lungs—that is, congesting them—instead of flowing out to the surface, and keeping the skin of a soft feel and a healthful warmth. Thus it is that the skin of all Consumptives has either a dry, hot feel, or a cold, clammy, dampness; at one time having cold chills creeping over them, causing them to shiver in the sun or hover over the fire; at another time, by the reaction, burning hot, the cheek a glowing red, the mouth parched with thirst. Another effect of the excess of acidity dissolving the albumen and carrying it into the blood is, that the blood is deficient in the fat, or oil, or carbon, which would have been made by the union of this albumen with alkaline secretions; the blood then wanting the fat or fuel which is necessary to keep the body warm, that which was already in the body, in the shape of what we call flesh, is used instead, and the man wastes away, just as when steamboat men, when out of wood, split up the doors, partitions, and other parts of the boat, to keep her going, she moves by consuming herself. So the Consumptive lives on, is kept warm by the burning up, the oxidation of his own flesh every day and every hour; this same wasting away being the invariable, the inseparable attendant of every case of true Consumption. He lives upon himself until there is no more fuel to burn, no more fat or flesh, and he dies—"nothing but skin and bone." What, then, must be done to cure a man of Consumptive disease?

He must be made more (what is called) "*fleshy*;" that is, he must have more fuel, fat, to keep him warm.

The acidity of the alimentary canal must be removed, in order that the food may be perfectly digested, so as to make pure blood, such as will flow healthfully and actively through every part of the system, and become congested, sluggish, stagnant nowhere.

To remove this acidity, the stomach must be made strong, and healthfully active; but no more than healthfully active, so as to convert the food into a substance fit for the manufacture of pure blood.

To make the stomach thus capable of forming a good blood material from the aliment introduced into it, as a perfect mill converts the grain into good flour or meal, there is behind the mill a power to turn it, there is behind the stomach powers to be exerted. These are the glandular system, the liver being the main one of all. This must be kept in healthful, operating order; if it acts too much or too little, the food is badly manufactured, and the blood which is made out of the food, and of the food alone, is imperfect and impure.

After all this is done, there is one more operation, which is the last finishing touch by which pure life-giving blood is made; ☞ a sufficient amount of pure air must come in contact with it before blood is constituted. This contact takes place in the lungs: not such a contact as the actual commingling of wine and water, for the air and what is soon to become blood are not mixed together; they are kept separate in different vessels. The air is in the lungs; that is, in the little bladders or cells, and this fluid, which is to be converted into blood, is in the little veins or tubes, which are spread around over the sides of the air-cells, as a vine is spread over a wall; but these little vessels have sides so very thin, that the life-giving material of the air passes through into the blood, just as the warmth of the sun passes through glass; but while this life-giving quality of the air passes into the blood, making it perfect, the impure and deathly ingredients of the blood pass out of it, into the air, which has just been deprived of its life. Thus it is, that while the air we draw in at a single breath is cool and pure and full of life, that which is expired is so hurtful, so poisonous, at least so destitute of life, that were it breathed in, instantly, uncombined with other air, by a perfectly healthy person, he would instantaneously die. So that pure air in breathing is most essentially indispensable; first, to impart perfection, life to the blood; and also to withdraw from it its death. No wonder, then, that a plentiful supply of pure air is so essential to the maintenance of health, so doubly essential to the removal of disease and restoration to a natural condition. No wonder, then, that when a man's lungs are decaying, and thus depriving him of the requisite amount of air, he so certainly fades away, unless the decay is first arrested, and the lung power or capacity restored.

The great principles, then, involved in the cure of Consumptive disease, or, professionally speaking, the great indications, are—

To cause the consumption and healthful digestion of the largest amount possible of substantial, nutritious, plain food.

To cause the patient to consume more pure air.

To bring about the first condition requires the exercise of extensive medical knowledge, combined with a wide experience and close and constant observation. To regulate healthfully the digestive apparatus—that is, to keep the whole glandular system of the human body in healthfully-working order—requires remedies and treatment as varied in their combinations almost as the varied features of the human face. Scarcely any two persons in a hundred are to be treated in the same way, unless you can find them of the same size, age, sex, constitution, temperament, country, climate, occupation, habits of life, and manner of inducing the disease. Here are ten characteristics which are capable, as every arithmetician knows, of a thousand different combinations; so that any person proposing any one thing as a remedy—a cure for Consumption, applicable to all cases and stages, must be ignorant or infamous beyond expression.

The two things above named will be always curative in proportion to their timely accomplishment. The ways of bringing these about must be varied according to constitution, temperament, and condition. The *mode* of doing the thing is not the essential, but *the thing done*. Beyond all question, the thing can be done: Consumption can be cured, and is cured in various ways. The scientific practitioner varies his means according to the existing state of the case. The name of the disease is nothing to him: he attacks the symptoms as they are at the time of prescribing; and if he be an experienced practitioner, he will know what ought to be done, and how it should be attempted, just as a classical scholar knows the meaning of a classical phrase or word the first time he ever sees it as perfectly as if he had seen it a thousand times before. And without setting myself up as an instructor to my medical brethren, I may here intimate my conviction, that the cure of Consumption would be a matter of every day occurrence, if they would simply study the nature of the disease, read not a word of how it had been treated by others, but observe closely every case, and treat its symptoms by general principles, as old as the hills, and follow up the treatment perseveringly, prescribe for the symptoms, and let the name and disease go. But then they must first understand perfectly the whole pathology of the disease—its whole nature. That, however, requires years of laborious study and patient observation.

The above things being true, as perhaps none will deny, it is worse than idle to be catching up every year some new medicine for the cure of Consumption. The readiness with which every new remedy is grasped at, shows beyond all question that the predecessors have been failures. Scores of cures have been eagerly experimented upon;—naphtha, cod liver oil, phosphate of lime, each will have its day, and each its speedy night, simply because no one thing can by any possibility be generally applicable, when solely relied upon. The physician must keep his eye steadily upon the thing to be done, varying the means infinitely, according to the case in hand. Therefore, the treatment of every individual case of Consumption must be placed in the hands of a scientific and experienced physician in time, and not wait, as is usually the case, until every balsam and syrup ever heard of has been tasted, tried, and experimented upon, leaving the practitioner nothing to work upon but a rotten, ruined hulk, leaving scarcely anything to do but to write out a certificate of burial, and receive as compensation all the discredit of the death.

The intelligent reader will perceive that I have spoken of the cure of Consumption as a matter of course. From the resolute vigor with which cod liver oil has been prescribed and (believingly) swallowed within a very few years past, one would suppose that almost every one believed that the cure of Consumption was a common every day affair. A few years ago, nobody thought so, except perhaps here and there a timid believer who kept his credence to himself, lest he should be laughed at. But the public got hold of the idea that cod liver oil was a remedy for the cure of Consumption, and swallowed thousands of barrels of what was said to be it, before they thought of inquiring for the facts of the case. I have never to this hour heard or read of a single case of true Consumption ever being perfectly and permanently arrested by the alone use of cod liver oil. No case that I have seen reported as cured would bear a legal investigation. There has always been some kind of reservation. It is my belief that all the virtues of cod liver oil, or any other oil, or phosphate of lime, as curative of consumption of the lungs, are contained in plain meat and bread, pure air and pure water; the whole of the difficulty being in making the patient competent to consume and assimilate enough of these. Herein consists the skill of the practitioner, and on this point he needs to bring to bear the knowledge, the study, the investigation, the observation, the experience of a life-time; and he who trusts to anything short of this, throws his life away.

The following articles are interesting and corroborative. "Littell's Living Age," No. 379, for August, the most popular and best conducted journal of the kind in America, copies from the London "Spectator" the following highly interesting and well-written article. Every line of it merits the mature consideration of the intelligent reader.

### "NEW HOSPITAL FOR DISEASES OF THE CHEST.

"While one-third of the deaths in the metropolis are ascribable to diseases of the chest, the hospital accommodation devoted to that class of diseases has heretofore been only one-tenth; that is to say, the most prevalent and destructive class of diseases has had the least counteraction among the poorer classes. This peculiar, if not studied neglect, must be ascribed to a notion, now happily dying out, that diseases connected with the respiratory organs, and especially the lungs, were virtually beyond the reach of certain or effective treatment. It was indifference to this old notion that Lord Carlisle made an admission, in his address to Prince Albert, on laying the first stone of the City of London Hospital for Diseases of the Chest—'We admit,' he said, 'that hospitals ought to give the preference to those maladies which afford a prospect of cure, rather than to those of a less hopeful character.' Now this admission, especially as compared with the qualification which followed it, that very much may be effected by precaution and a timely counteraction, is far too strong for the truth. Without accepting as literally true the inference of a physician eminent in the treatment of pectoral diseases, that all persons are at one time or other visited by maladies of that class, we believe it is certain that the proportion of mortality, enormous as it is, scarcely represents the comparative extension of such diseases. In the practical and popular sense of the word, it may be said that cure is as common in the class of pectoral diseases as in any other class. It has become much more common, indeed, since the great advance that has been made with the knowledge of such complaints in our own day. This advance has been of a two-fold character. The immense progress of physiological inquiry has thrown great light on the connection and common causes of most cognate diseases, not only with each other but with the general health, and has thus enormously augmented the power of the physician in treating them by medicine and regimen. The invention of the stethescope, by placing the exploration of the inner chest within reach of observation, has given a distinctness of knowledge on the most characteristic and dangerous symptoms, heretofore unattainable: it has thus completed the round of evidence which establishes the connection of diseases, and at the same time guides the nature and application of topical treatment.

In discovering that the prevalency of pectoral diseases was far greater than had been supposed, science has also discovered how much more they are under subjection to the general laws of physiology and medicine. This branch of science, however, is younger than others—a fact which teaches us to remember how much is to be expected from the active and vigorous intellects now devoted to its exploration. We may also remember that while the primary object of hospitals is the relief of sufferers who are too poor to obtain it for themselves, they are also great instruments for the benefit of society at large, by checking the inroads of disease where it could not otherwise be encountered. They are still more signally valuable as great schools for the study of the diseases to which they are appropriated. They exemplify most powerfully the double blessing of charity, for him that gives as well as him that receives; the aid extended by a hospital to the poor is returned to the rich in the

